# Comparison Between In-Person and Audio-Only Virtual Prenatal Visits and Perinatal Outcomes

**DOI:** 10.1001/jamanetworkopen.2021.5854

**Published:** 2021-04-14

**Authors:** Elaine L. Duryea, Emily H. Adhikari, Anne Ambia, Catherine Spong, Donald McIntire, David B. Nelson

**Affiliations:** 1 Department of Obstetrics and Gynecology, University of Texas Southwestern Medical Center, Dallas; 2 Department of Obstetrics and Gynecology, Parkland Health and Hospital System, Dallas, Texas

## Abstract

**Importance:**

Ensuring access to prenatal care services in the US is challenging, and implementation of telehealth options was limited before the COVID-19 pandemic, especially in vulnerable populations, given the regulatory requirements for video visit technology.

**Objective:**

To explore the association of audio-only virtual prenatal care with perinatal outcomes.

**Design, Setting, and Participants:**

This cohort study compared perinatal outcomes of women who delivered between May 1 and October 31, 2019 (n = 6559), and received in-person prenatal visits only with those who delivered between May 1 and October 31, 2020 (n = 6048), when audio-only virtual visits were integrated into prenatal care during the COVID-19 pandemic, as feasible based on pregnancy complications. Parkland Health and Hospital System in Dallas, Texas, provides care to the vulnerable obstetric population of the county via a high-volume prenatal clinic system and public maternity hospital. All deliveries of infants weighing more than 500 g, whether live or stillborn, were included.

**Exposures:**

Prenatal care incorporating audio-only prenatal care visits.

**Main Outcomes and Measures:**

The primary outcome was a composite of placental abruption, stillbirth, neonatal intensive care unit admission in a full-term (≥37 weeks) infant, and umbilical cord blood pH less than 7.0. Visit data, maternal characteristics, and other perinatal outcomes were also examined.

**Results:**

The mean (SD) age of the 6559 women who delivered in 2019 was 27.8 (6.4) years, and the age of the 6048 women who delivered in 2020 was 27.7 (6.5) years (*P* = .38). Of women delivering in 2020, 1090 (18.0%) were non-Hispanic Black compared with 1067 (16.3%) in 2019 (*P* = .04). In the 2020 cohort, 4067 women (67.2%) attended at least 1 and 1216 women (20.1%) attended at least 3 audio-only virtual prenatal visits. Women who delivered in 2020 attended a greater mean (SD) number of prenatal visits compared with women who delivered in 2019 (9.8 [3.4] vs 9.4 [3.8] visits; *P* < .001). In the 2020 cohort, 173 women (2.9%) experienced the composite outcome, which was not significantly different than the 195 women (3.0%) in 2019 (*P* = .71). In addition, the rate of the composite outcome did not differ substantially when examined according to the number of audio-only virtual visits attended.

**Conclusions and Relevance:**

Implementation of audio-only virtual prenatal visits was not associated with changes in perinatal outcomes and increased prenatal visit attendance in a vulnerable population during the COVID-19 pandemic when used in a risk-appropriate model.

## Key Points

**Question:**

Were audio-only virtual prenatal visits during the COVID-19 pandemic associated with a change in perinatal outcomes in a vulnerable population?

**Findings:**

In this cohort study of 12 607 women, 173 women (2.9%) experienced placental abruption, stillbirth, cord pH less than 7.0, or full-term neonatal intensive care unit admission, which was not significantly different than the 195 women (3.0%) affected in 2019. The rate of this composite outcome also did not differ significantly when stratified by the number of virtual prenatal visits.

**Meaning:**

In this study, women who delivered in 2020 following implementation of audio-only prenatal virtual visits did not experience more adverse pregnancy outcomes than women who delivered in 2019.

## Teaser

This cohort study compares perinatal outcomes among women who received in-person pernatal visits in 2019 vs those who received audio-only virtual prenatal visits during the COVID-19 pandemic in 2020.

## Introduction

The importance of prenatal care has been recognized for more than a century.^1^ Use of prenatal care is associated with decreased maternal mortality, preterm birth, neonatal death, and stillbirth.^1,2,3,4,5^ Ensuring access to prenatal care services has been challenging, with 5% of women in the US entering prenatal care late in pregnancy or not at all.^6^ Disparities in prenatal care access have been identified in the US, with women identifying as Black or Hispanic less likely to participate in prenatal care. For example, 23% of non-Hispanic Black women and 18% of Hispanic women received inadequate prenatal care in 2016, compared with only 11% of non-Hispanic White women.^6^ This discrepancy is further realized in the disparate maternal mortality rates in minority populations in the US.^7^ Despite national recognition of the problem and emphasis on improving care access among women of color and vulnerable populations, disparities persist and may be exacerbated by events such as the COVID-19 pandemic.^8,9^

Before the COVID-19 pandemic, prenatal care in the US was largely delivered via in-person visits, with telehealth encounters limited to delivery of ancillary services such as smoking cessation, nutritional education, and lactation counseling.^10,11^ With the onset of the pandemic, many institutions provided modified prenatal care using innovative platforms such as drive-through clinics and telehealth visits with varied results.^11,12,13,14,15,16^ Telehealth visits may use synchronous video or audio-only technology, with each modality having both benefits and limitations.^17^ Despite a lack of supporting data, video technology has de facto been considered superior to audio-only visits. Before the pandemic, state and federal regulatory requirements did not support the use of an audio-only platform.^17,18^ Video visits require both the clinician and patient to possess specific hardware, internet bandwidth, and technological literacy.^15^ Given that 50% of women in the US rely on Medicaid for coverage of prenatal and delivery services, a significant portion may not be able to adequately participate in prenatal care using video-based platforms.^6,9,18,19,20,21^

In a previous study on the incorporation of audio-only virtual visits, the patient responses were positive.^22^ As we have continued to use audio-only prenatal visits during the ongoing COVID-19 pandemic, our primary aim was to examine the clinical associations, if any, between audio-only virtual prenatal visits and perinatal outcomes. A secondary aim was to examine the association between multiple virtual prenatal visits and perinatal outcomes. We hypothesized that adverse perinatal outcomes were not increased in 2020 following integration of audio-only prenatal virtual visits compared with 2019, when traditional in-person prenatal care was standard.

## Methods

This cohort study compared perinatal outcomes among women who delivered at Parkland Hospital, Dallas, Texas, between May 1 and October 31, 2019, with those who delivered between May 1 and October 31, 2020, when synchronous audio-only virtual prenatal visits had been adopted. The study was approved by the University of Texas Southwestern Medical Center Institutional Review Board, and a waiver of informed consent was granted owing to determination of minimal risk. This study followed the Strengthening the Reporting of Observational Studies in Epidemiology (STROBE) reporting guideline for cohort studies.

Parkland Hospital serves the population of women with medical indigence of Dallas County and operates a neighborhood-based, administratively and medically integrated public health care system for pregnant women. All women delivering at Parkland Hospital were enrolled in care at a neighborhood clinic for antenatal and postpartum care continuity. Women with medical or pregnancy complications are referred to a centrally located maternal-fetal medicine clinic, which is a high-risk prenatal clinic staffed by maternal-fetal medicine faculty and fellows from the University of Texas Southwestern Medical Center. Medical management of both routine and high-risk pregnancy is provided throughout the health care system using standardized protocols. Women delivering in 2019 received traditional in-person prenatal care, with no option for virtual prenatal visits.

In March 2020, to address social distancing needs while continuing to provide prenatal care during the COVID-19 pandemic, we rapidly implemented audio-only virtual prenatal visits. Synchronous audio-only visit types were chosen based on the resources immediately available. Virtual prenatal visits were incorporated into the prenatal care schedule at specific intervals, based on guidance form the World Health Organization and the American College of Obstetricians and Gynecologists, with in-person visits coinciding with laboratory testing, vaccinations, and sonography (Table 1).^14,23^ For women in the maternal-fetal medicine clinic, a similar schedule for virtual visits was used when feasible and deemed safe and acceptable by the faculty physicians on an individual patient basis with regard to the specific pregnancy complications present and the need for concurrent laboratory tests, measurement of vital signs, and physical examinations, such as cervical examination. Therefore, not all women, especially those at later gestational ages, were recommended for virtual prenatal care, although no specific medical condition was universally considered a contraindication to virtual care. A certified interpreter service was used to facilitate all visits with women for whom English was not their primary language, both for in-person and virtual visits.

**Table 1.** Prenatal Visit Schedule Incorporating Virtual Visits^a^

Approximate weeks of gestation	Type of visit	Comment
10	In-person	Initial prenatal visit, to include initial obstetric laboratory testing
14	Virtual	None
18-20	In-person	Ultrasonographic and maternal serum screening performed
24	In-person	Glucose tolerance testing performed
28	In-person^b^	If Rho(D) immune globulin administration required
32	In-person	Third trimester laboratory studies performed
34	Virtual	None
36	In-person	Gonorrhea/chlamydia testing performed
37	Virtual	None
38	In-person	None
39	In-person	None
40	In-person	None
41	In-person	None

^a^ Adapted from Holcomb et al.^22^

^b^ On July 6, 2020, the laboratory testing and injections associated with the 24-, 28-, and 32-week visits were consolidated to coincide at the 28-week visit, in an effort to improve use of virtual visits.

Maternal demographic characteristics, visit data, and perinatal outcomes of women after delivery were obtained from an obstetric quality database, which contains data extracted from the electronic medical record by dedicated research nurses according to standard protocols and definitions contained within a manual of operations and routinely validated with cross-checks. Maternal race/ethnicity was defined by the patient as documented within the electronic medical record, with any woman with an ethnicity of Hispanic grouped as such; all other women were subsequently grouped by race. Identification and analysis of race and ethnicity was performed to facilitate comparison of the 2 cohorts and their baseline characteristics. All deliveries of infants weighing more than 500 g, whether live or stillborn, were included. Operative vaginal deliveries are limited to forceps delivery at our institution. The diagnosis of gestational hypertension, which includes any form of hypertension in pregnancy that does not predate pregnancy, preeclampsia with severe features, and placental abruption was based on clinician documentation. Major malformations were recorded based on the neonatal physical examination and imaging studies, and according to pediatric clinician documentation. Clinical indications for interventions such as cesarean delivery or neonatal intensive care unit admission did not change during the study period, with care dictated by established treatment protocols. The primary outcome studied was a composite of placental abruption, stillbirth, neonatal intensive care unit admission in a full-term (≥37 weeks) infant, and umbilical cord blood pH less than 7.0. This composite was chosen based on the availability of high-quality data on each outcome and the frequent use of each finding in the literature as a perinatal outcome measure.

### Statistical Analysis

Maternal characteristics and perinatal outcomes were compared between women delivering in the 2 cohorts. Based on data from 2017-2018, 2.9% of women experienced at least 1 component of the primary composite outcome. Anticipating no change in the rate of the composite in 2019, a sample size of at least 5413 women per cohort would be able to detect a one-third increase in the composite outcome with a power of 80% and a type I error of 5%. Thus, cohorts of 5413 women would allow for detection of an increase in the primary composite outcome from 2.9% to 3.9%. Pearson χ^2^ analysis was used for categorical outcomes and a 2-tailed, unpaired *t* test was used for continuous outcomes and the Mantel-Haenszel test for trend was applied as indicated. Effect sizes were measured as relative risk (RR) with 95% CI for the χ^2^, difference in means with 95% CI for the *t* test, and Hodges-Lehmann estimate of shift difference for the median with 95% CI. Differences with a *P* value <.05 were considered significant. All statistical analyses were performed using SAS, version 9.4 (SAS Institute Inc).

## Results

We studied 6559 women (mean [SD] age, 27.8 [6.4] years) who delivered in 2019 and 6048 women (mean [SD] age, 27.7 [6.5] years) who delivered in 2020 (*P* = .38) (Table 2). Of women delivering in 2020, 1090 (18.0%) were non-Hispanic Black compared with 1067 (16.3%) in 2019 (*P* = .04). Women delivering in 2020 gained slightly more weight during their pregnancy (mean [SD], 9.8 [6.1] vs 9.3 [5.8] kg; *P* < .001). Although statistically significant, this difference was not considered to be clinically relevant. Because Parkland Hospital serves as a county-supported public health system, approximately 86% of deliveries were funded by Medicaid or the Children’s Health Insurance Program, 8% were self-pay or free care, and only 6% were funded by commercial insurance during the study period, with payer status stable between 2019 and 2020. The rates of multiple gestation, gestational or pregestational diabetes, and chronic hypertension were not substantially changed.

**Table 2.** Maternal Characteristics of Women Delivering in 2019 vs 2020^a^

Maternal characteristic	No. (%)		*P* value
	Audio-only virtual prenatal visits (May 1, 2020-October 31, 2020)	Traditional prenatal care (May 1, 2019-October 31, 2019)	
No. of women	6048	6559	
Maternal age, mean (SD), y	27.7 (6.5)	27.8 (6.4)	.38
Race/ethnicity			
Hispanic	4568 (75.5)	5060 (77.1)	.04
Non-Hispanic			
Black	1090 (18.0)	1067 (16.3)	
White	216 (3.6)	219 (3.3)	
Other	171 (2.8)	213 (3.2)	
Nulliparous	1769 (29.2)	1953 (29.8)	.52
BMI, mean (SD)			
At delivery	33.2 (6.6)	32.9 (6.4)	.01
At first encounter	29.4 (6.7)	29.3 (6.7)	.46
Multiple gestation	91 (1.5)	88 (1.3)	.44
Diabetes			
Gestational	448 (7.4)	462 (7.0)	.43
Pregestational	107 (1.8)	129 (2.0)	.41
Chronic hypertension	273 (4.5)	293 (4.5)	.90

Abbreviation: BMI, body mass index (calculated as weight in kilograms divided by height in meters squared).

^a^ Traditional prenatal care was conducted for women delivering from May 1 to October 31, 2019. Audio-only virtual prenatal visits were conducted for women delivering from May 1 to October 31, 2020.

Women presented earlier for prenatal care in 2020, with a median gestational age at the first prenatal visit of 11 weeks (interquartile range [IQR], 8-17 weeks) in 2020 vs 12 weeks (IQR, 9-18 weeks) in 2019 (*P* < .001). In addition, women delivering in 2020 had a greater mean (SD) number of prenatal encounters, including both virtual and in-person, compared with women delivering in 2019 (9.8 [3.4] vs 9.4 [3.8] visits; *P* < .001). Women accessed prenatal care before delivery with similar frequency in 2020 and 2019, with 6395 women (97.5%) in 2019 and 5906 women (97.7%) in 2020 attending at least 1 visit before delivery. In the 2020 cohort, 4067 women (67.2%) completed at least 1 audio-only virtual prenatal visit and 1216 women (20.1%) completed 3 or more audio-only virtual prenatal visits.

In the 2020 cohort, there were 173 women (2.9%) who experienced the composite outcome of placental abruption, stillbirth, neonatal intensive care unit admission in a full-term (≥37 weeks) infant, or umbilical cord blood pH less than 7.0, which was not significantly different from the 195 women (3.0%) in 2019 (*P* = .71) (Table 3). When examining other obstetric outcomes in 2020 vs 2019, there was no significant difference in the frequency of gestational hypertension (1147 [19.0%] vs 1320 [20.1%]; *P* = .10), preeclampsia with severe features (649 [10.7%] vs 697 [10.6%]; *P* = .85), or preterm birth (<37 weeks: 593 [9.8%] vs 672 [10.2%]; *P* = .41). Women in 2020 were slightly more likely to undergo cesarean delivery than women in 2019 (RR, 1.06; 95% CI, 1.01-1.12), although this difference was no longer significant after adjusting for race and body mass index at delivery. Shoulder dystocia was less common in 2020 compared with 2019 (RR, 0.49; 95% CI, 0.26-0.92), and this difference remained significant after adjustment (adjusted RR, 0.48; 95% CI, 0.26-0.91). The rates of postpartum hemorrhage and peripartum hysterectomy were not significantly different, although women were less likely to require a transfusion during or following delivery in 2020 compared with 2019 (adjusted RR, 0.84; 95% CI, 0.70-0.99). The median maternal length of stay at delivery was 4 days (IQR, 3-4 days) for both cohorts; however, when examined with the Hodges-Lehmann estimate of shift difference, this duration was overall shorter in 2020 (*P* < .001). Median gestation age at delivery was 39 weeks (IQR, 38-40 weeks) in both cohorts (*P* = .12).

**Table 3.** Perinatal Outcomes for Women in 2019 vs 2020^a^

Perinatal outcome	No. (%)		*P* value	RR (95% CI) [IQR]	Adjusted RR (95% CI) [IQR]^b^
	Audio-only virtual prenatal visits	Traditional prenatal care			
No. of women	6048	6559			
Composite outcome^c^	173 (2.9)	195 (3.0)	.71	0.96 [0.79-1.18]	0.96 [0.78-1.17]
Stillbirth	29 (0.5)	40 (0.6)	.32	0.79 [0.49-1.27]	0.80 [0.50-1.29]
Full-term NICU admission	94 (1.6)	98 (1.5)	.78	1.04 [0.79-1.38]	1.03 [0.78-1.36]
Placental abruption	40 (0.7)	56 (0.9)	.21	0.77 (0.52-1.16)	0.76 (0.51-1.14)
Arterial cord gas pH <7.0	17 (0.3)	22 (0.4)	.64	0.86 [0.46-1.62]	0.82 [0.44-1.55]
Gestational hypertension	1147 (19.0)	1320 (20.1)	.10	0.94 (0.88-1.01)	0.93 (0.86-0.99)
Preeclampsia with severe features	649 (10.7)	697 (10.6)	.85	1.01 (0.91-1.12)	0.99 (0.89-1.09)
Preterm birth, wk					
<37	593 (9.8)	672 (10.2)	.41	0.96 (0.86-1.06)	0.96 (0.86-1.06)
<34	202 (3.3)	203 (3.1)	.44	1.08 [0.89-1.31]	1.09 (0.90-1.31)
Mode of delivery					
Vaginal, spontaneous	4046 (66.9)	4476 (68.2)	.11	0.98 (0.96-1.00)	0.99 (0.96-1.01)
Vaginal, forceps assisted	82 (1.4)	119 (1.8)	.04	0.75 (0.57-0.99)	0.75 (0.57-0.99)
Cesarean delivery	1920 (31.7)	1964 (29.9)	.03	1.06 (1.01-1.12)	1.03 (0.98-1.09)
Primary cesarean	816 (13.5)	788 (12.0)	.01	1.12 (1.02-1.23)	1.10 (1.01-1.21)
Postpartum hemorrhage^d^	570 (9.4)	580 (8.8)	.26	1.07 (0.95-1.19)	1.04 (0.93-1.16)
Need for transfusion	216 (3.6)	279 (4.3)	.049	0.84 (0.71-1.00)	0.84 (0.70-0.99)
Hysterectomy	13 (0.2)	26 (0.4)	.07	0.54 (0.28-1.05)	0.53 (0.27-1.04)
Shoulder dystocia	14 (0.2)	31 (0.5)	.02	0.49 (0.26-0.92)	0.48 (0.26-0.91)

Abbreviations: IQR, interquartile range; NICU, neonatal intensive care unit; RR, relative risk.

^a^ Traditional prenatal care was conducted for women delivering from May 1 to October 31, 2019. Audio-only virtual prenatal visits were conducted for women delivering from May 1 to October 31, 2020.

^b^ Adjusted for body mass index at delivery and race/ethnicity.

^c^ Women with multiple gestations were included in the composite if any fetus or newborn was affected.

^d^ Defined as estimated blood loss greater than 1000 mL.

In 2019, 40 stillborn infants (6 per 1000) were delivered, and the rate did not increase significantly in 2020 (30 infants [5 per 1000]; *P* = .39). The rate of major malformations was unchanged, at 1.9% of deliveries in both cohorts (*P* = .99). When examining neonatal outcomes in live-born infants without major malformations, 304 of 6483 (4.7%) infants in 2019 required admission to the neonatal intensive care unit, compared to 296 of 5997 (4.9%) in 2020 (*P* = .52). The rate of fetal acidemia was also unchanged, with an umbilical cord blood pH less than 7.0 noted in 22 infants in 2019 and 15 infants in 2020 (*P* = .41). As in 2019, total hospital days for infants was shorter in 2020, although the median and IQR were not different for the 2 cohorts (3 days [IQR, 3-4 days]; *P* < .001).

No deleterious outcome was found when evaluating outcomes according to the number of audio-only virtual visit encounters in 2020 (Table 4). Women with a greater number of virtual visits were less likely to experience placental abruption (*P* = .01 for trend), deliver prematurely (*P* < .001 for trend), or require transfusion at delivery (*P* = .005 for trend). Infant outcomes, including stillbirth and major malformations, were equivalent in women who attended a greater number of audio-only virtual prenatal visits.

**Table 4.** Perinatal Outcomes Stratified by Number of Audio-only Virtual Visits Performed in 2020

Variable	Traditional prenatal care, No. (%)	Audio-only virtual prenatal visits, No. (%)				*P* value for trend
		0	1	2	≥3	
No. of women	6559	1981	1612	1239	1216	NA
Composite outcome^a^	195 (3.0)	66 (3.3)	47 (2.9)	35 (2.8)	25 (2.1)	.15
Stillbirth	40 (0.6)	17 (0.9)	2 (0.1)	5 (0.4)	5 (0.4)	.08
Full-term NICU admission	98 (1.5)	28 (1.4)	28 (1.7)	23 (1.9)	15 (1.2)	.87
Placental abruption	56 (0.9)	19 (1.0)	13 (0.8)	5 (0.4)	3 (0.2)	.01
Arterial cord gas pH <7.0	22 (0.4)	7 (0.4)	4 (0.3)	2 (0.2)	4 (0.4)	.56
Preeclampsia with severe features	697 (10.6)	235 (11.9)	174 (10.8)	120 (9.7)	120 (9.9)	.31
Preterm birth, wk						
<37	672 (10.2)	259 (13.1)	147 (9.1)	88 (7.1)	99 (8.1)	<.001
<34	203 (3.1)	108 (5.5)	49 (3.0)	25 (2.0)	20 (1.6)	.003
Cesarean delivery	1964 (29.9)	633 (32.0)	516 (32.0)	372 (30.0)	399 (32.8)	.07
Postpartum hemorrhage^b^	580 (8.8)	187 (9.4)	150 (9.3)	115 (9.3)	118 (9.7)	.30
Need for transfusion	279 (4.3)	90 (4.5)	49 (3.0)	40 (3.2)	37 (3.0)	.005
Hysterectomy	26 (0.4)	2 (0.1)	4 (0.2)	3 (0.2)	4 (0.3)	.37
Shoulder dystocia	31 (0.5)	5 (0.3)	3 (0.2)	3 (0.2)	3 (0.2)	.06
All delivered infants, No.	6647	2020	1633	1252	1235	NA
Major malformation	124 (1.9)	43 (2.1)	40 (2.4)	20 (1.6)	10 (1)	.09
Stillbirth	40 (0.6)	17 (0.8)	3 (0.2)	5 (0.4)	5 (0)	.11
Live-born infants without major malformations, No.	6483	1960	1590	1227	1220	NA
NICU admission	304 (4.7)	123 (6.3)	82 (5.2)	44 (3.6)	47 (4)	.12
Umbilical cord gas pH <7.0	22 (0.4)	6 (0.3)	3 (0.2)	2 (0.2)	4 (0.4)	.48

Abbreviations: NA, not applicable; NICU, neonatal intensive care unit.

^a^ For women with multiple gestations, they are included in the composite if any fetus or newborn was affected.

^b^ Defined as estimated blood loss greater than 1000 mL.

## Discussion

Women who delivered in 2020 following implementation of audio-only prenatal virtual visits did not experience more adverse perinatal outcomes compared with women who delivered in 2019. Furthermore, access to care in a population with medical indigence increased with the use of audio-only virtual prenatal visits, which was associated with a greater attendance rate of virtual visits compared with in-person appointments.^22^ Large-scale, rapid deployment of virtual prenatal care was feasible across 10 community-based prenatal clinics, with the use of synchronous audio-only virtual prenatal visits.

Our finding that a composite outcome of placental abruption, neonatal intensive care unit admission among full-term (≥37 weeks) infants, or umbilical cord blood pH less than 7.0 was not increased among women accessing prenatal care with integrated, synchronous audio-only visits may provide reassurance to both patients and obstetricians. Our findings are in line with those of Stowe et al,^24^ who reported no increase in stillbirth during the COVID-19 pandemic lockdown in England. We expanded the analysis by including detailed pregnancy characteristics and a comparison of other uncommon but meaningful obstetric and neonatal outcomes. Randomized trials using telehealth to deliver other ancillary services, such as smoking cessation or nutrition counseling, in obstetric patients have shown mixed results.^10^ Butler Tobah et al^25^ reported high obstetric patient satisfaction with an enhanced model of integrated phone or online communication that included home monitoring devices and online support resources; however, obstetric and neonatal outcomes were not measured. Similarly, a previous study reported patient satisfaction with audio-only virtual care, and now provides an expansion of obstetric outcomes analyzed over 6 months with this care model expanded from the one used by Holcomb et al.^22^ Our study suggests both feasibility in large-scale implementation of a prenatal care model with integrated audio-only visits and acceptability of this model of care in terms of major maternal and neonatal outcomes. The evidence provided herein suggests that synchronous audio-only virtual visits should be considered a legitimate type of telehealth visit outside of the setting of a pandemic, based on both patient satisfaction and obstetric and neonatal outcomes in an inner-city public hospital.

### Strengths and Limitations

Strengths of our study include the ability to compare uncommon but meaningful adverse pregnancy outcomes in a cohort of over 6000 women for whom prenatal and delivery care is largely covered by Medicaid. Despite potential challenges with telemedicine, use of audio-only virtual visits ensured that 67.2% of women delivering in 2020 attended at least 1 virtual prenatal visit and 97.7% of women accessed prenatal care before delivery.

The study is limited by the retrospective nature of the examination, although our large sample sizes and universal ascertainment of deliveries may reduce potential selection bias in our primary analysis. Only perinatal outcomes were examined, and we cannot comment whether there was a delay in diagnoses, for example, during prenatal care that did not ultimately affect outcomes. For women with high-risk medical conditions or pregnancy complications, virtual visits were assigned on a patient-level basis by faculty physicians in the maternal-fetal medicine clinic. We acknowledge selection bias for women with fewer high-risk conditions to receive more virtual prenatal care; for example, these women were also less likely to experience preterm birth. However, this limitation represents a consideration for implementation of virtual care in obstetrics: our results cannot be applied to a protocol in which women receive virtual prenatal care regardless of concurrent medical diagnoses and clinical context. Access to risk-appropriate prenatal care—whether in-person or virtual—is necessary for satisfactory perinatal outcomes. Just as our institution placed prenatal clinics in areas to facilitate access to care for women delivering at our hospital, the delivery of virtual prenatal care must be tailored to the patient.

## Conclusions

Given the rapid implementation of virtual prenatal care across the country during the COVID-19 pandemic, we believed it was important to measure the use of a new care model in our high-volume, public maternity center. Audio-only virtual visits provided necessary and complete care in a population without ready access to the resources and technologic literacy necessary to engage in video visits. Despite this, we face a dilemma: audio-only virtual prenatal visits are currently deemed acceptable as emergency measures, but not as complete visits for the sake of reimbursement. Our findings suggest safety, efficacy, and legitimacy of a synchronous, audio-only virtual prenatal care platform and advocate for equity in reimbursement in parallel to in-person visits.
